# Inhibition of CD34+ cell migration by matrix metalloproteinase-2 during acute myocardial ischemia, counteracted by ischemic preconditioning

**DOI:** 10.12688/f1000research.9957.3

**Published:** 2017-02-06

**Authors:** Dominika Lukovic, Katrin Zlabinger, Alfred Gugerell, Andreas Spannbauer, Noemi Pavo, Ljubica Mandic, Denise T. Weidenauer, Stefan Kastl, Christoph Kaun, Aniko Posa, Inna Sabdyusheva Litschauer, Johannes Winkler, Mariann Gyöngyösi

**Affiliations:** 1 Department of Cardiology, Medical University of Vienna, Vienna, Austria

**Keywords:** acute myocardial infarction, stem cell mobilization, preconditioning, SDF-1/CXCR4 axis, MMP-2

## Abstract

**Background.** Mobilization of bone marrow-origin CD34+ cells was investigated 3 days (3d) after acute myocardial infarction (AMI) with/without ischemic preconditioning (IP) in relation to stromal-derived factor-1 (SDF-1α)/ chemokine receptor type 4 (CXCR4) axis, to search for possible mechanisms behind insufficient cardiac repair in the first days post-AMI. **Methods.** Closed-chest reperfused AMI was performed by percutaneous balloon occlusion of the mid-left anterior descending (LAD) coronary artery for 90min, followed by reperfusion in pigs. Animals were randomized to receive either IP initiated by 3x5min cycles of re-occlusion/re-flow prior to AMI (n=6) or control AMI (n=12). Blood samples were collected at baseline, 3d post-AMI, and at 1-month follow-up to analyse chemokines and mobilized CD34+ cells. To investigate the effect of acute hypoxia, SDF-1α and matrix metalloproteinase (MMP)-2 *in vitro *were assessed, and a migration assay of CD34+ cells toward cardiomyocytes was performed. **Results. **Reperfused AMI induced significant mobilisation of CD34+ cells (baseline: 260±75 vs. 3d: 668±180; P<0.001) and secretion of MMP-2 (baseline: 291.83±53.40 vs. 3d: 369.64±72.89; P=0.011) into plasma, without affecting the SDF-1α concentration. IP led to the inhibition of MMP-2 (IP: 165.67±47.99 vs. AMI: 369.64±72.89; P=0.004) 3d post-AMI, accompanied by increased release of SDF-1α (baseline: 23.80±12.36 vs. 3d: 45.29±11.31; P=0.05) and CXCR4 (baseline: 0.59±0.16 vs. 3d: 2.06±1.42; P=0.034), with a parallel higher level of mobilisation of CD34+ cells (IP: 881±126 vs. AMI: 668±180; P=0.026), compared to non-conditioned AMI. *In vitro*, CD34+ cell migration toward cardiomyocytes was enhanced by SDF-1α, which was completely abolished by 90min hypoxia and co-incubation with MMP-2. **Conclusions**. Non-conditioned AMI induces MMP-2 release, hampering the ischemia-induced increase in SDF-1α and CXCR4 by cleaving the SDF-1α/CXCR4 axis, with diminished mobilization of the angiogenic CD34+ cells. IP might influence CD34+ cell mobilization via inhibition of MMP-2.

## Introduction

Heart regeneration after ischemic insult is still a matter of debate in spite of extensive research conducted in this field. One of the endogenous cardiac repair mechanisms is the mobilization of regenerative cells derived from bone marrow (BM), followed by migration and homing of the cells in the ischemic myocardial tissue ^1^. Several factors have been identified that play a role in the mobilization of BM-origin stem and progenitor cells, and assist in migration and homing, such as chemotactic factors, complement fractions, cytokines, microRNAs or microvesicles. Among these substances, the axis of the stromal-derived factor-1 alpha [SDF-1α; chemokine receptor 12 (CXCL12)] and chemokine receptor type 4 (CXCR4) exerts the strongest chemoattractant stimulus for migration and homing of cells in the BM and tumors, but also in ischemic tissues, such in case of myocardial ischemia or ischemic stroke ^2^. The local upregulation of SDF-1α attracts the cells covered with CXCR4 receptors towards the SDF-1α gradient, facilitating the migration of the cells into the target organ tissues.

Exploiting the beneficial effect of the SDF-1α/CXCR4 axis in cardiac repair has been performed by repeated injections of granulocyte-colony stimulating factor (G-CSF) applied in patients, which aims to release the stem and premature cells from BM and activate the cellular CXCR4 expression of the reparative cells by interrupting the BM-SDF-1α/CXCR4 axis ^3^. However, despite the enhanced cell release and migration, and stimulation of the endogenous cardiac progenitor cells, the efficacy of clinical cardiac cell-based therapy in patients with recent acute myocardial infarction (AMI) led to ambiguous results ^4^, especially if the regenerative cell therapy was performed very early after ischemic injury ^5^.

Among several mechanisms explaining the cardiac regenerative processes, secretion of distinct chemokines, cytokines, and growth factors may play an important role in the course of events of myocardial infarction ^2^. Tang *et al* demonstrated upregulated SDF-1α expression in infarcted mouse myocardial tissue after implantation of mesenchymal stem cells simulated by vascular endothelial growth factor (VEGF). This led to increased mobilisation of BM-derived stem cells ^6^. Also, the upregulation of pro-inflammatory cytokines, such tumor necrosis factor (TNF)α ^7^ and interleukin (IL)-8 ^8^, might initiate processes triggering increased cell trafficking, since myocardial infarction is associated with the inflammatory response ^8^. In contrast, matrix metalloprotease (MMP)-2 cytokine is known to be the inhibitor of SDF-1α, implicating its inactivation ^9^. Another mechanistic process is cardioprotection, induced by either ischemic pre-, post- or remote conditioning, or by release of numerous cardioprotective substances, but their clinical importance is still doubtful. Ischemic preconditioning (IP) has been shown to exhibit cardioprotective mechanisms, and stimulates the recruitment and homing of progenitor cells toward ischemic myocardium in the early phases of cardioprotection in several animal models ^7^. IP-induced cardioprotection possesses bi-phasic effect by initiation of protective mechanisms in the early phase appearing within the first 3 hours following myocardian insult ^10^ and in the late phase, which re-appear 24 hours and lasts for 72 hours ^11^. In our study, we focused on the late window of cardioprotection in relation with stem cell mobilisation and cytokine releases.

In our present experiment, we have investigated the mobilization of BM-origin CD34+ cells 3 days after reperfused AMI in relation to the SDF-1α/CXCR4 axis in a clinically relevant *in vivo* pig model. We conducted the experiments in domestic female pigs, since the female pigs tolerate the ischemic burden better regarding mortality as compared to male pigs ^12^.

We measured the release of several cytokines, such as MMP-2, VEGF, fibroblast growth factor (FGF)-2, IL-8 and TNFα, to investigate and explain the possible mechanisms behind insufficient cardiac repair in the first days post-AMI. In addition, the current study explored possible additional benefits elicited by IP that involve release of cytokines, CD34+ cells, and MMP-2 expression.

## Methods

### Design of the porcine closed-chest reperfused acute myocardial infarction (AMI)

**Figure 1.**
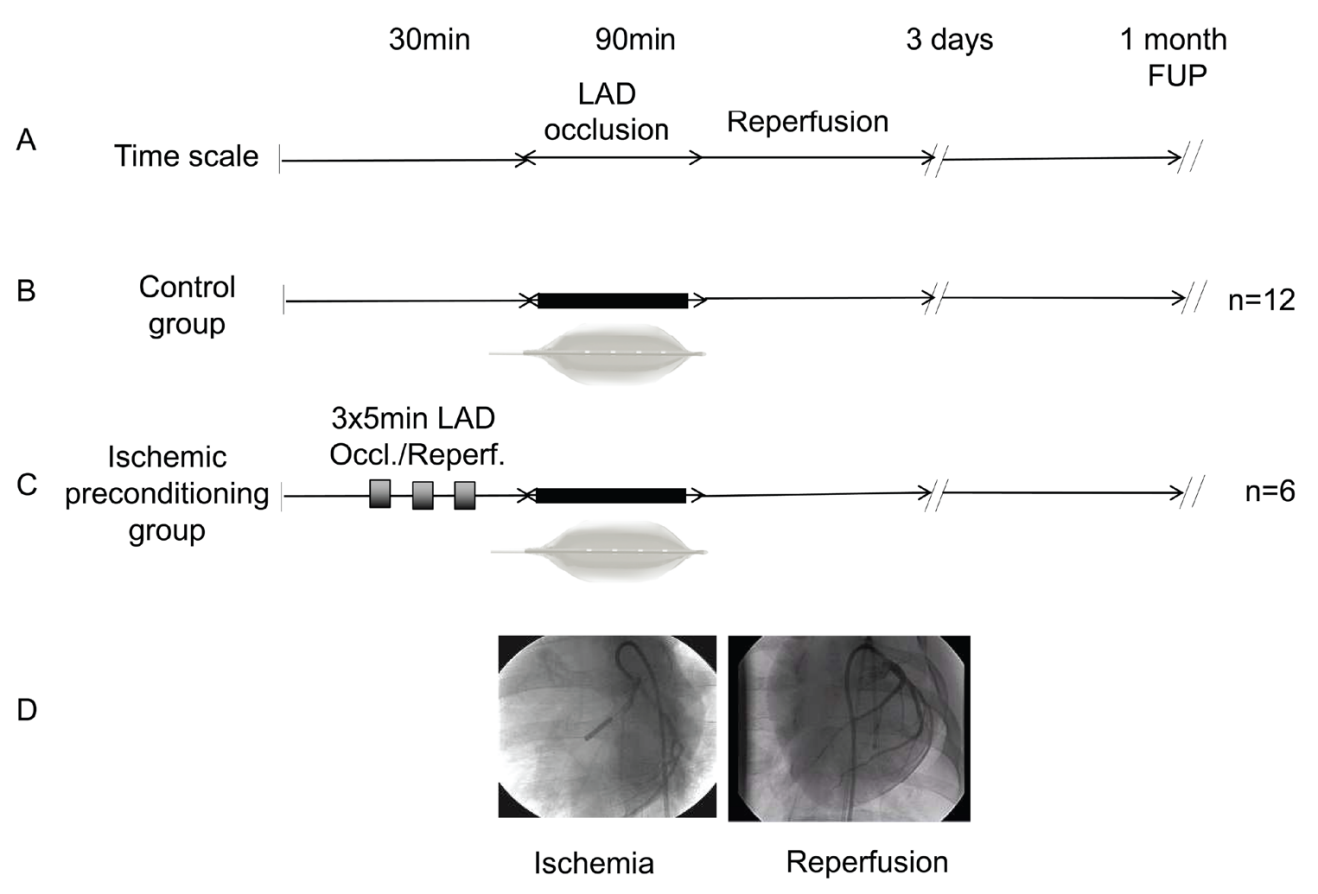
Design of the porcine closed-chest reperfused myocardial infarction experiments. ( **A**) Time scale: 30min anaesthesia, followed by 90min occlusion of the mid left anterior descending coronary artery (LAD), followed by reperfusion. Follow-up (FUP) times 3 days and 1 month. ( **B**) Control group (n=12). ( **C**) Ischemic preconditioning group (n=6) was induced by 3×5min cycles of ischemia/reperfusion (balloon inflation/deflation) prior to 90min balloon occlusion of the LAD. ( **D**) Angiographic pictures of the balloon occlusion of the LAD (left, left anterior oblique acquisition at 45°) and control angiography after restoration of the reperfusion (right, anteroposterior view).

Randomly selected female domestic pigs (n=21; weight, 30–35kg) underwent percutaneous coronary intervention (PCI) under general anaesthesia in order to perform either ischemic preconditioning (group IP; n=6) or non-conditioned AMI (group control; n=12), with a block 1:2 randomisation, due to expected higher mortality in the control group. Animals in both groups underwent 90min percutaneous balloon occlusion of the left anterior descending (LAD) coronary artery at the origin of the first diagonal branch following reperfusion (balloon deflation). IP was initiated prior to 90min LAD occlusion by 3×5min repetitive cycles of artery re-occlusion and reperfusion. One pig in the IP and two animals in the control group died during the AMI intervention, all remaining animals (n=6 in IP and n=12 in control group) survived for 1 month after the experimental procedure ( Figure 1).

### Porcine closed-chest model of ischemia/reperfusion

All procedures were performed with the approval of the local Experimental Animal Care Committee (EK SOI/31/26-11/2014) of the University of Kaposvar, Hungary, conforming to the *Guide for the Care and Use of Laboratory Animals* published by the US National Institute of Health (NIH Publication No. 85–23, revised 1996). All animal experiments were conducted at the institute of diagnostic imaging and radiation oncology, University of Kaposvar.

Female domestic pigs received 12mg/kg ketamine, 1mg/kg xylazine and 0.04mg/kg atropine as anaesthesia. The anaesthesia was deepened via mask maintaining 1.5–2.5 vol % isofluran, 1.6–1.8 vol % O _2_ and 0.5 vol % N _2_O. In total, 200IU/kg of heparin was administrated via the right femoral artery, and selective angiography of LAD arteries was performed prior to induction of myocardial ischemia (MI). MI was induced by 90min balloon occlusion (3.0mm ø, 15mm length, 5atm; Maverick, Boston Scientific, MA, USA) at the mid-part of the LAD artery following balloon deflation. The % O _2_ saturation, blood pressure and electrocardiogram were continuously measured during the intervention.

### Blood sampling

Blood samples were collected from the femoral vein for the detection of biological markers. Samples were centrifuged at 2000×g for 10min, and the plasma and serum samples were stored at -20°C until the analysis was performed. For fluorescent activated cell sorting (FACS) analysis, whole blood was collected into EDTA-treated tubes (BD Vacutainer®; Becton, Dickinson and Company, New Jersey, USA) at baseline, 3 days post MI and 1 month follow-up (FUP). All blood samples were processed within 6h.

### Enzyme linked immunosorbent assay (ELISA)

Plasma level of stromal cell-derived factor-1 (porcine SDF-1α ELISA Kit; Neoscientific, Germany), chemokine (C-X-C motif) receptor 4 (pig CXCR4 ELISA Kit; Abbexa, UK), 72kDa isoform of matrix metalloproteinase-2 protein (porcine MMP-2 ELISA Kit; MyBioSource, CA, USA), fibroblast growth factor-2 (porcine FGF-2 ELISA Kit; Neoscientific, Germany), and vascular endothelial growth factor (porcine VEGF ELISA Kit; Neoscientific, Germany) were detected using commercial ELISA kits, according to the manufacturer’s instructions. Tumor necrosis factor alpha (Porcine TNFα Quantikine ELISA Kit; R&D Systems, MN, USA), and interleukin-8 (pig IL-8 ELISA Kit; Abcam, UK) were detected from serum, according to the manufacturer’s instructions.

Absorbance readings at wavelength 450nm were performed on the automated plate reader VIKTOR3 (Perkin Elmer, MA, USA), and the resulting values were determined by interpolation from a standard curve. Measurements were performed in duplicates. Plasma or serum levels of markers were measured at baseline, 3d post MI and at 1 month FUP.

### FACS analysis of CD34+ cells in peripheral blood

FACS analysis of whole blood samples was performed at baseline, 3d post MI and at 1 month FUP in order to address the kinetics of mobilized CD34+ cells *in vivo*. EDTA-treated venous blood samples (100μl) were labelled with PE-DY647-conjugated CD34+ antibody (monoclonal antibody; host/isotype: mouse/IgG1; cat# MA1-19770; Thermo Fisher Scientific, Waltham, MA, USA) or the corresponding isotype control (PE-conjugated mouse IgG1; cat# MA1-10415; Thermo Fisher Scientific, Waltham, MA) for 20min at room temperature. Anti-human CD34+ antibody was utilized due to lack of commercially available porcine-specific CD34+ marker (dilution: 5μl antibody/100μl whole porcine blood). Subsequently, erythrocyte cell lysis was performed, according to the manufacturer’s protocol, using Dako-Uti Lyse ^TM^ (Dako, Agilent Technologies, Santa Clara, CA, USA) following fixation with PBS containing 1% paraformaldehyde. FACS analysis was performed on CyFlow ^®^ ML/space flow cytometer (Sysmex Partec, Görlitz, Germany) with acquired 100.000 events within the gated region of mononuclear cells of forward versus side scatter. Absolute counts of CD34+ cells were obtained by multiplying the ratio of the CD34+ cells obtained in the flow cytometry analysis and absolute count of leucocytes per 1μl of blood.

### Isolation of human adult cardiac myocytes

Human adult cardiac myocytes (HACMs) were isolated from the left ventricular tissue obtained from the hearts of patients undergoing heart transplantation. Mechanical dissociation of the tissue and separation of the cardiomyocytes from fibroblasts detached to Petri-dish surface was performed, as described previously ^13^. All tissue donors gave their informed written consent to the study. The study was approved by the local ethical committee (Medical University of Vienna, Austria; EK 151/2008) and complies with the Declaration of Helsinki.

Human cord blood CD34 positive cells (CD34+ cells) were purchased from StemCell Technologies Company (Grenoble, France). The cells were used in in vitro cell migration assay to assess their migratory capacity toward HACMs. Since porcine CD34+ cells are not commercially available, we used human cardiomyocytes and human cord blood CD34+ cells.

### Cell migration assay

**Figure 2.**
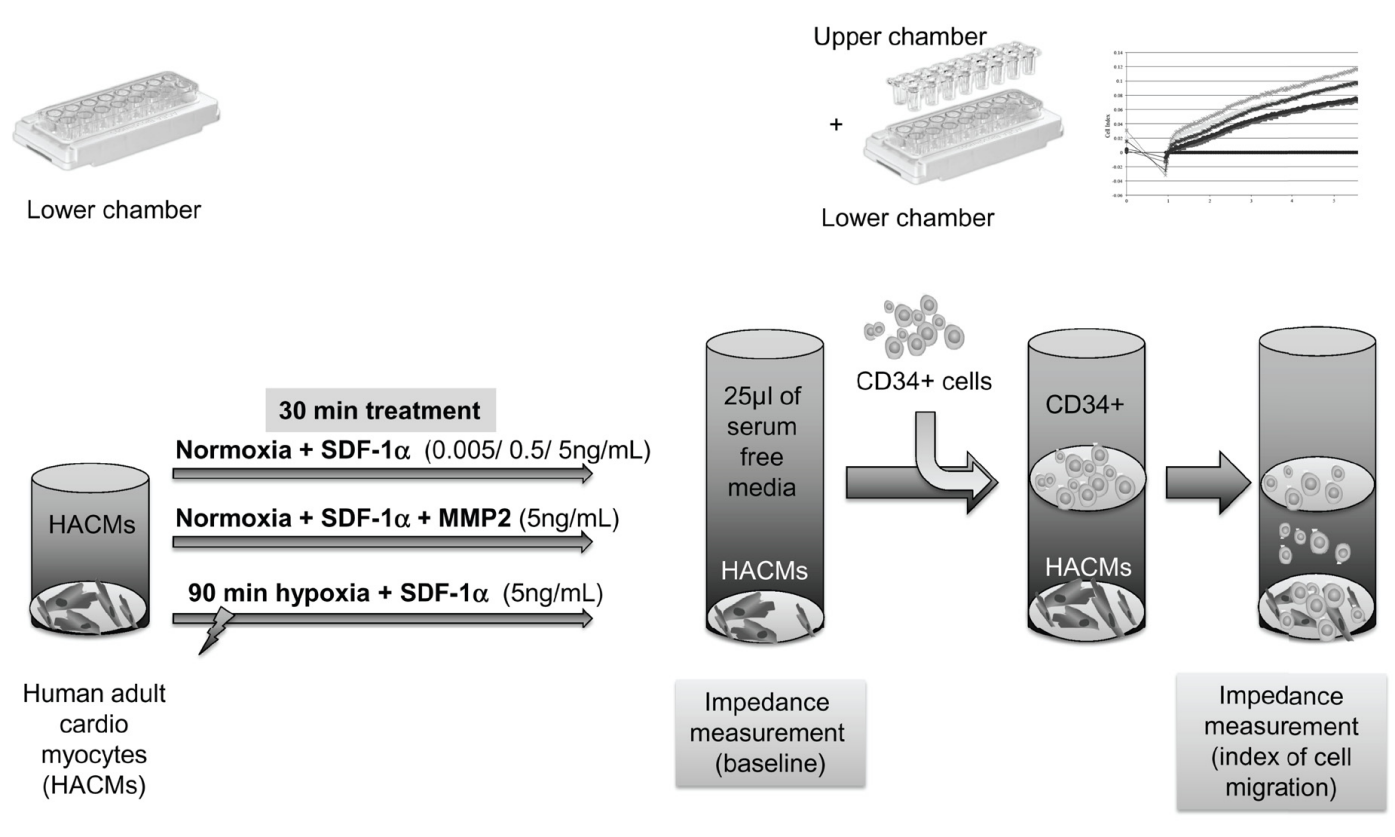
Design of the cell migration assay. Human adult cardiac myocytes (HACMs) were incubated in the lower chamber. After 30min incubation with different treatments: either adding stromal-derived factor-1-alpha (SDF-1α) in increasing concentrations, or adding the highest concentration of SDF-1α and matrix-metalloprotease-2 (MMP-2), or the cells were kept under hypoxia for 90min, followed by the addition of SDF-1α. Lower and upper chamber were then combined, and after adding serum-free media to the upper chamber, baseline impedance measurements were performed, followed by adding CD34+ cells. Impedance measurements were performed to quantify the CD34+ cell migration towards HAMCs.

Migration of CD34+ cells was monitored by commercially available Roche xCELLigence System (Acea Bioscience, CA, USA), according to the manufacturer’s instructions. Briefly, 160μl suspension of HACM cells (conc. 10.000 cells/well) was resuspended in M199 cardiac cell culture media (Sigma-Aldrich, Vienna, Austria) containing 20% FBS and 1% Pen/Strep solution (Gibco™, Thermo Fischer Scientific, MA, USA). Cell suspension was transferred to the lower chamber of the CIM-Plate with integrated gold microelectrode sensors ( Figure 2).

HACMs were incubated under normoxic conditions with increasing doses (0.005, 0.5 and 5.0ng/mL) of SDF-1α (Sigma-Aldrich, Vienna, Austria) to evaluate the maximal SDF-1α chemoattractant effect on CD34+ cells towards HCAMs.

In order to analyse the effect of MMP-2 and hypoxia on the mobilisation of CD34+ cells toward HCAMs, HCAMs were incubated with SDF-1α (with the elaborated maximal effect of 5.0ng/mL) either with co-incubation with MMP-2 (5.0ng/mL; Sigma-Aldrich, Vienna, Austria) under normoxia, or under 90min hypoxic conditions.

Hypoxia (90min, 37°C, 1% O _2_) was induced in HCAM cell culture on the CIM-Plate by sealing the cell culture plate in an airtight plastic bag (Microbiology Anaerocult® IS Bag; Merck Millipore, Vienna, Austria) containing a dry anaerobic indicator strip.

 In total, 25μl serum-free medium (M199 containing 0.1% FBS) was added to the upper chamber 30min after treatments with the various substances, and the chambers were combined for background measurements. Subsequently, CD34+ cells (100.000 cells/well) were transferred to the upper chamber of the CIM-Plate with polyethylene terephthalate membrane (PET) with 8μm pore diameter and measurements were repeated. Migrated cells translocated through the PET-membrane and changed the impedance signal captured by sensors in the lower chamber. The background was subtracted from all results and each experiment was repeated three times ( Figure 2).

### Statistical analysis

Continuous parameters were expressed as means ± standard deviation. The effects between the groups and within the groups (baseline vs. 3d post-AMI) were analyzed by two-way analysis of variance (ANOVA) with repeated measures model with Bonferroni correction. The mean differences between the groups were detected by independent Student’s *t*-test. Differences were considered statistically significant at P<0.05. Statistical analyses were performed with SPSS software (version 17.0; Macintosh; SPSS IBM).

## Results

### AMI-induced cytokine release and CD34+ mobilization

**Figure 3.**
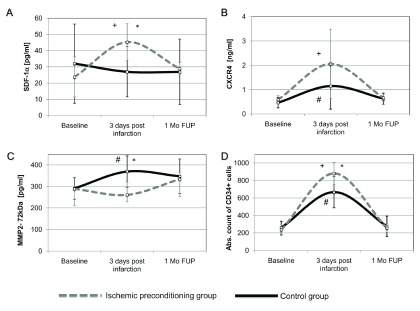
Time-dependent changes of plasma SDF-1α, CXCR4 and MMP-2 (72kDa), and CD34+ cell count in IPC and AMI group. Plasma concentrations of circulating ( **A**) SDF-1α; ( **B**) CXCR4; ( **C**) MMP-2 (72kDa) protein isoform measured by porcine-specific ELISAs. ( **D**) Absolute count of circulating CD34+ cells were determined by FACS analysis. Concentrations are expressed as mean± standard deviation. *P<0.05 between the IP and control group; +P<0.05 between baseline and 3 day values within the IP group; #P<0.05 between baseline and 3 day values within the control (non-conditioned AMI) group. SDF-1α, stromal-derived factor-1 alpha; CXCR4, C-X-C motif chemokine receptor 4; MMP-2 (72kDa), matrix metalloproteinase-2, 72kDa isoform; IPC, ischemic preconditioning; AMI, acute myocardial infarction; 1 Mo FUP, 1 month follow-up.

Reperfused AMI did not enhance the release of SDF-1α early (at 3 days) post-AMI (baseline: 32.02±24.35 vs. 3 days post-AMI: 26.97±15.43pg/ml; P=0.41) ( Figure 3). In contrast, the circulating level of CXCR4 (baseline: 0.47±0.22 vs. 3 days post-AMI: 1.15±0.95ng/ml; P=0.034) significantly increased at 3 days post-AMI with concomitant induced mobilization of CD34+ cells (baseline: 260±75 vs. 3 days post-AMI: 668±180cells/μl; P<0.001). However, the level of MMP-2 was increased significantly (baseline: 291.83±53.40 vs. 3 days post-AMI: 369.64±72.88pg/ml; P=0.011), which might explain the cleaved SDF-1α/CXCR4 axis ( Figure 3).

**Figure 4.**
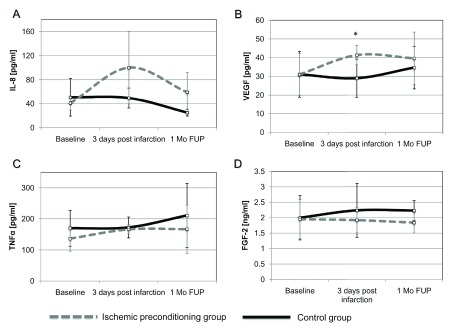
Time-dependent changes of plasma FGF-2, VEGF, serum IL-8 and TNFα analysed in IPC and AMI group. Plasma concentrations of circulating ( **A**) IL-8, ( **B**) VEGF, ( **C**) TNFα, ( **D**) FGF-2 Concentrations are expressed as mean± standard deviation. *P<0.05 between the IP and control group. FGF-2, fibroblast growth factor-2; VEGF, vascular endothelial growth factor; IL-8, interleukin-8; TNFα, tumor necrosis factor alpha; IPC, ischemic preconditioning; AMI, acute myocardial infarction; 1 Mo FUP, 1 month follow-up.

The circulating levels of the angiogenic cytokines (FGF-2, VEGF, IL-8 and TNFα) were not changed significantly at 3-days post-AMI in the AMI group ( Figure 4A–D).

### IP induces CD34+ cell mobilisation via CXCR4/SDF-1α axis

IP led to the significantly higher stimulation of SDF-1α chemokine release with its putative receptor, CXCR4, into circulation, accompanied by downregulation of MMP-2. The number of CD34+ cells significantly increased as compared to the animals in the control group (non-conditioned AMI).

The plasma level of SDF-1α significantly increased 3 days post infarction in the IP group as compared to control AMI group (IP: 45.29± 11.31 vs. control: 27.00±15.43pg/ml; P=0.037), with normalization at the 1-month FUP (IP: 28.87± 3.81 vs. control: 26.91± 20.24pg/ml; P=0.85) ( Figure 3A). Enhanced SDF-1α secretion was accompanied by significant increase of its soluble CXCR4 receptor after 3 days post-AMI (baseline: 0.59±0.16 vs. 3 days post-AMI: 2.06±1.42ng/ml; P=0.034); however, this did not reach statistical significance between the groups (IP: 2.06 ±1.42 vs. control: 1.15 ±0.95ng/ml; P=0.79) ( Figure 3B).

IP significantly downregulated the secretion of MMP-2 into plasma at 3 days FUP as compared to the control AMI group (IP: 165.67±47.99 vs. control: 369.64±72.89pg/ml; P=0.004), which returned to the baseline level at the 1-month FUP control (IP: 334.00±93.10 vs. control: 347.58±80.47pg/ml; P=0.074) ( Figure 3C).

FACS analysis was performed to reflect the impact of chemoattractant release on cell migration. We observed a significant parallel increase of mobilized CD34+ cells in both non-conditioned AMI and IP groups 3 days post infarction (IP: 881±126 vs. control: 668±180cells/μl; P=0.026, returning to the baseline level after 1 month FUP (IP: 255±50 vs. control: 275±118cells/μl; P=0.85) ( Figure 3D).

### IP-induced changes in the levels of circulating cytokines

A trend towards increase of IL-8 was observed in the IP group at 3 days and 1 month post infarction (IP: 100.18±60.42 vs. control: 49.52±16.68pg/ml; P=0.055) at day 3 and (IP: 59.32±32.88 vs. control: 25.19±5.76pg/ml; P=0.059) at 1 month ( Figure 4A).

The concentration of VEGF in plasma significantly increased in IP group at day 3 post infarction as compared with controls (IP: 41.35±5.12 vs. control: 29.01± 10.18pg/ml; P=0.021) ( Figure 4B).

IP did not affect the changes in serum concentrations of TNFα as compared to the control group ( Figure 4C).

 The plasma level of FGF-2 was not significantly changed at day 3 by IP as compared to the control group (IP: 1.90±0.41 vs. control: 2.22±0.88ng/ml; P=0.45) ( Figure 4D).

### Inhibition of SDF-1α-induced CD34+ cell migration by MMP-2 *in vitro*

**Figure 5.**
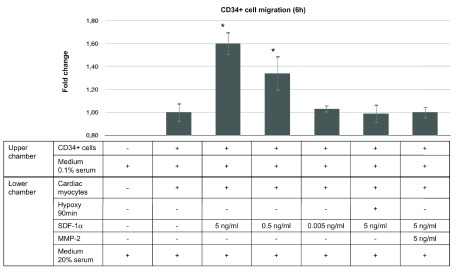
CD34+ cell migration toward human adult cardiomyocytes culture under different conditions. Migration was quantified as fold change impedance compared to the baseline conditions. Adding of SDF-1α in different concentrations induced chemotaxis of the CD34+ cells in a dose-dependent manner. A total of 90min hypoxia followed by a change of the medium eliminated the chemotactic effect of SDF-1α, and blocked CD34+ cell migration. This migration effect of SDF-1α was similarly eliminated if MMP-2 was added to the normoxic cell culture. Depicted results express impedance values measured at 6h post-treatment. Background results were subtracted from each impedance measurement. Parameters are expressed as mean ± standard deviation. Each experiment was repeated three times. *P<0.001 compared to the baseline normalized value. SDF-1α, stromal derived factor-1α; MMP-2, matrix metalloproteinase-2.

In order to prove the oppositional effect of MMP-2 on CD34+ cell mobilisation, we added MMP-2 to cultured HACMs, stimulated with SDF-1α, and quantified the CD34+ cell migration towards the HACMs ( Figure 5).

SDF-1α treatment stimulated the migration of CD34+ cells toward HACMs under normoxic conditions in a dose-dependent manner. The maximal chemotactic effect (1.6± 0.11 fold change; P<0.001) was achieved by adding 5ng/ml concentration of SDF-1α, while 0.5ng/mL and 0.05ng/mL SDF-1α resulted in a migration rate of 1.35± 0.18 fold (P<0.001) and 1.08±0.02 fold change (P=0.43), respectively, compared to the control HACMs and CD34+ cells culture without SDF-1α.

Co-incubation of HACMs with MMP-2 under normoxic conditions completely eliminated the SDF-1α chemotactic effect to CD34+ cell migration towards the cardiomyocytes (0.98±0.1 fold change; P=0.71).

Interestingly, incubation of the cardiomyocytes under 90min hypoxic conditions inhibited migration of CD34+ cells, even if the highest effective dose of SDF-1α (5ng/ml) was added to the cell culture as a chemoattractant (1.00±0.04 fold change; P=0.75).

Raw data for XCelligence measurements of cell migration assay Click here for additional data file. Copyright: © 2017 Lukovic D et al. 2017 Data associated with the article are available under the terms of the Creative Commons Zero "No rights reserved" data waiver (CC0 1.0 Public domain dedication).

Raw data obtained from ELISA and FACS analyses Click here for additional data file. Copyright: © 2017 Lukovic D et al. 2017 Data associated with the article are available under the terms of the Creative Commons Zero "No rights reserved" data waiver (CC0 1.0 Public domain dedication).

## Discussion

Here, we demonstrate that 1) myocardial ischemia triggers the release of circulating MMP-2, which inhibits SDF-1α and CXCR4 release; 2) SDF-1-induced migration of CD34+ cells towards cardiomyocytes was inhibited by MMP-2 *in vitro*; 3) IP inhibited MMP-2 release, thereby increasing both SDF-1α and CXCR4 levels, resulting in a higher level of CD34+ cell mobilization 3 days post ischemic injury in *in vivo* condition; 4) IP induced VEGF secretion in the second window of cardioprotection.

### AMI leads to MMP-2 release

Reperfused AMI led to an increase in CXCR4, but not SDF-1α, at 3 days post-infarction, with moderate enhancement of circulating CD34+ counts. Similarly, AMI caused significant elevation of MMP-2, produced by macrophages in case of acute tissue injury. MMP-2 disrupts the SDF-1α/CXCR4 axis, by cleaving SDF-1α to N-terminally truncated SDF-1 ^14^. This form of SDF-1 is unable to trigger CXCR4 signalling and prevent the chemoattractant function of SDF-1α/CXCR4 in human progenitor cells ^15^. Since the increased upregulation of MMP-2 post-AMI may inhibit retention of hematopoietic stem cells in the ischemic injury site, targeted modulation of MMP-2 expression has potential to improve outcome of regenerative therapies ^9^.

### IP induces CD34+ mobilisation by SDF-1α/CXCR4 axis *in vivo*

Our previous study demonstrated that IP in early phase post-infarction (early window of protection, 2h after reperfusion start) induced mobilization of BM-derived haematopoietic (HSCs) and mesenchymal stem cells (MSCs) involving the release of distinct cytokines ^7^. In our present work, we analyzed the effect of IP on the mobilization of CD34+ regenerative cells and measured the cytokine release (MMP-2, VEGF, FGF-2, IL-8 and TNFα) in the late (second) window of protection.

In contrast with the non-conditioned AMI group, we observed significantly elevated SDF-1α plasma level in the IP group at 3 days post infarction, as compared to the AMI group. This confirmed our earlier assumptions that SDF-1α is released in a later time window after IP ^7^. Previous *in vitro* and *in vivo* experiments have shown an increased cell migration ability responding to treatment with SDF-1α ^16^ or increased mobilisation of BM-derived cells toward injured tissue after SDF-1α overexpression ^6, 17^. The putative receptor for SDF-1α chemokine is CXCR4, which is expressed also in mouse cardiomyocytes ^17^ and mobilises mesenchymal stem cells in the ST-segment elevation of myocardial infarction patients ^2^. The elevated level of SDF-1α was paralleled by an increased number of circulating CD34+ cells. This suggests that IP stimulates CD34+ cell migration by SDF-1α/CXCR4 upregulation within the first days after AMI.

The increased concentration of MMP-2 (72kDa) at 3 days post-infarction was completely abolished by IP, which might be an additional beneficial effect of IP in a translational large animal model, and is similar to mice experiments ^18^. IP has shown cardioprotective effects against ischemia/reperfusion injury in accepted experimental models. Induction of IP in a mouse model led to improvement of cardiac function and increasing cell survival, accompanied by release of BM- derived cells ^10^. Accordingly, our previous ^4^ and present study suggest that IP stimulates endogenous mechanisms, promoting the recruitment of CD34+ cells in both early and late windows of cardioprotection.

### MMP-2 and hypoxia abolish SDF-1 *α*-induced CD34+ cell mobilisation *in vitro*

In order to prove the direct confounding effects of MMP-2 on SDF-1α/CXCR4, we have performed *in vitro* experiments, and observed that MMP-2 completely inhibited SDF-1α -induced CD34+ cell mobilization.

Interestingly, our experiments also revealed that 90min hypoxia abolishes the SDF-1α chemotactic effect *in vitro*. By contrast, it has been reported that hypoxia inducible factor 2, which is released in hypoxia, binds to the promotor sequence of CXCR4, the SDF-1α putative receptor, and activates the migratory activity of the endothelial progenitor cells ^19^. We cannot completely explain our findings, but we assume that the release of hypoxia-triggered factors, such as MMP-2, may locally inhibit the migratory capacity of the regenerative cells. This is also in concordance with the findings in humans; early administration of regenerative cells has debatable effects on myocardial regeneration ^20^.

### Effect of AMI and IP on FGF-2, VEGF, IL-8 and TNFα release

Non-conditioned AMI did not influence the release of circulating cytokine FGF-2, VEGF, IL-8 and TNFα. In the first 3 days post-AMI. Our findings are similar to the study of Husebye *et al* ^21^ reporting no increase in TNF-alpha and IL-8 levels in patients with STEMI and randomized to placebo group. In contrast, IP induced a marked release of circulating VEGF and a trend towards increase in IL-8 3 days post-AMI, indicating the stimulation of additional pro-migratory cytokines by IP for enhanced cardioprotection. IL-8 is a pro-inflammatory C-X-C chemokine that is also involved in activation of pro-angiogenic processes and re-introduction of progenitor cells into the circulation. The study of Schomig *et al.* demonstrated significantly increased IL-8 level in AMI patients as compared to patients diagnosed with stable angina ^8^. In our study, we observed a trend toward increased release of IL-8 in the clinically relevant porcine reperfused “STEMI” model. The levels of CXCR4 increased both in controls (with AMI) and IP groups, with a trend towards higher increase in the IP group 3-day post AMI. The differences between our and other studies might be explained by the pre- and peri-AMI medication of patients with standard care that may contribute to changes in plasma levels of cytokines in AMI patients ^8^. Results of plasma cytokine levels would be more informative if it measured more often, in an extended time window. The area under the curve (AUC) calculation of the cytokine release data might have delivered additional results. However, for a simple blood sampling, the animals must have been fully anaesthesized, which procedure signifies an additional stress for the animals with recent AMI with predicted higher mortality.

In our previous experiments, IP induced the release of VEGF plasma levels immediately after myocardial infarction (first window of protection) ^7^. In the present experiment, VEGF was still increased 3 days post AMI in the IP group (second window of protection) as compared to the control AMI group. Similarly to our study, Kamota *et al.* showed an amplified secretion of VEGF and SDF-1α up until 6 hours post infarction in a mouse model of IP ^10^. Tang *et al.* also reported induced mobilisation of stem cells by VEGF/ SDF-1α trafficking in a rat model ^6^.

FGF-2 is an important chemotactic factor and it is also a prominent cardioprotective and angiogenic agent ^22^. Since FGF-2 was not significantly induced by IP in our experiment, we assume that this protein did not participate in mechanisms of IP-elicited late window of cardioprotection.

Acute phase of AMI after IP is characterized by an increased level of TNFα triggering a release of additional cytokines, such as IL-6, IL-8, and cell adhesion molecules. Our previous data demonstrated that IP resulted in elevated levels of TNFα in serum with concomitant IL-8 increase immediately after reperfusion induction ^7^. A later time window after AMI revealed heterogeneous results. TNFα remained moderately increased 3 days post infarction with continuous moderate increase after 1 month FUP in both groups, most probably due to developing chronic phase of myocardial infarction. Interestingly, IP induced a trend towards enhanced IL-8 release, which is a potent progenitor cell mobilisation enhancer responding to ischemia, although it is also associated with pro-inflammatory processes ^8, 9^.

In conclusion, the present study revealed that AMI induces MMP-2 release, which hampered the ischemia-induced increase in SDF-1α and CXCR4 by cleaving the SDF-1α/CXCR4 axis. This led to diminished mobilization of the angiogenic CD34+ cells. IP induced CD34+ cell mobilization in the late phase (second window), thereby also increasing circulating SDF-1α and CXCR4, parallel with enhanced VEGF secretion. One mechanism of this beneficial effect of IP might be the inhibition of AMI-induced MMP2-release. *In vitro* migration assay confirmed the anti-migratory effect of MMP-2 and the direct negative association of MMP-2 and SDF-1α-induced cell migration. Accordingly, our experiment might explain the inhibited homing of mobilized or transplanted cells in the ischemic myocardium resulting in decreased efficacy of cell-based therapies early after AMI.

### Limitations

Even though we demonstrate IP-induced mobilisation of CD34+ cells in a large animal model of reperfused AMI, the clinical relevance of IP remains uncertain. We have concentrated on mechanisms involved in cell mobilisation in terms of chemokine and cytokine secretion.

An important limitation is the utilisation of human CD34+ FACS antibody due to lack of commercially available porcine products. However, the number of mobilized CD34+ cells correspond with the available mobilized cell numbers published several times ^2, 10, 23^; bearing in mind, that the normal count of white blood cells of pigs is 12–20 thousand cells/μl blood.

We revealed one additional possible beneficial mechanism of IP, namely the inhibition of MMP-2 release with consequent higher mobilization of CD34+ cells, which was confirmed in our *in vitro* experiment. However, a direct association between IP - MMP-2 - CD34+ axis had to be confirmed *in vivo*, by blocking MMP2 in animals subjected to AMI and IP. We have not measured myocardial MMP-2 level, which analysis would require harvesting of the animals maximal 72h post IP-AMI (second window of protection), and our animals survived 1-month follow-up.

We have chosen female pigs for the experiments because of a clear gender differences observed in female and male rodents, rabbits, dogs and pigs ^24^; the incidence of cardiogenic shock and life-threatening arrhythmias were more frequent in male than female pigs by using the closed-chest reperfused AMI model ^12^.

We are aware, that serial blood sampling would have given more information, e.g. the evident changes in mobilisation of bone marrow-derived cells following myocardial infarction occur at day 3, 7 and 14 post-ischemia ^25^. However, we have focused on the second window of protection, which ends at day 3.

Inclusion of a sham-operated control group would promote better comparison of the results. However, we decided to not supplement the study groups with control group of animals without infarction with expected constant baseline level of biomarkers. Indeed, we intended to apply the “reduction” principle of the 3R (reduction, refinement and replacement) concept regarding the *in vivo* experiments. 

Isolation of human cardiomyocytes was performed from samples obtained from both female and male patients. Since the cells were utilized for *in vitro* experimental evaluation, the “gender” of the cells in the cell culture was not relevant for the *in vivo* experiments.

## Data availability

The data referenced by this article are under copyright with the following copyright statement: Copyright: © 2017 Lukovic D et al.

Data associated with the article are available under the terms of the Creative Commons Zero "No rights reserved" data waiver (CC0 1.0 Public domain dedication). 

**Dataset 1**. Raw data for XCelligence measurements of cell migration assay (DOI: 10.5256/f1000research.9957.d142079 ^26^).

**Dataset 2**. Raw data obtained from ELISA and FACS analyses (DOI: 10.5256/f1000research.9957.d142080 ^27^).
