# Supplementary material for: Inhibition of CD34+ cell migration by matrix metalloproteinase-2 during acute myocardial ischemia, counteracted by ischemic preconditioning
Source: F1000Res. 2017 Feb 6;5:2739. Originally published 2016 Nov 22. [Version 3] doi: 10.12688/f1000research.9957.3 (PMC5321121; doi:10.12688/f1000research.9957.3)
Supplement: Peer review history

# Peer review: Referee response for version 3

**DOI:** 10.5256/f1000research.11610.r19943

**Published:** 2017-02-20

**Authors:** Amadou K.S. Camara

**Affiliations:** 1 Department of Anesthesiology, Medical College of Wisconsin (MCW), Milwaukee, WI, USA

General comments:

 This is revised manuscript following my first appraisal of the work. In my view, the reviewers have responded very well to most of my previous concerns. However, some minor concerns are still present.

 Specific comments:

 Introduction (page 3)

 First paragraph, “…migration and homing of cells in the BM and tumors, but also in ischemic tissues, such in case of myocardial ischemia…” Revise to read, “…migration and homing of cells in the BM and tumors, but also in ischemic tissues, as in the case with myocardial ischemia…”

 Third paragraph, “In contrast, matrix…(MMP)-2 cytokine is known to be the inhibitor of…” Revise to read, “In contrast, matrix…(MMP-2) cytokine is known to be an inhibitor of…”

 Fourth paragraph, “…in the early phase appearing within the first 3 hours following myocardian insult….” Replace “myocardian” with “myocardial”.

 Methods:

 Page 5 (Isolation of human adult cardiac myocytes)

 “Mechanical dissociation of the tissue and separation of the cardiomyocytes from fibroblasts detached to…” Replace “detach to” with detach “from”.

 Results:

 Page 7 (IP induces CD34+ cell mobilization via CXCR4/SDF-1 alpha axis)

 Third paragraph, “IP significantly dowregulated the secretion of…” Revise to read, “IP significantly reduced the secretion of…”  

 Discussion:

 Page 9 (Effect of AMI and IP on…release)

 First paragraph, “In the first 3 days post-AMI.” What else? Last three sentences of this paragraph are unclear. The sentence “Results of plasma cytokine levels…in an extended time window” should be revised. “The area under the curve (AUC)…” It is unclear what the authors are referring to here. "However, from a simple blood sampling…., which procedure signifies an additional stress…with recent AMI with predicted higher mortality”. The meaning of this sentence is unclear, especially the phrase “which procedure signifies…”

 Lastly, in my previous comment in 3C, (Cell migration), the authors provided a description of why the 90 min hypoxia was conducted in the in vitro model, but this information is missing in the body text of the manuscript. Why is this the case?

I have read this submission. I believe that I have an appropriate level of expertise to confirm that it is of an acceptable scientific standard.

# Peer review: Referee response for version 3

**DOI:** 10.5256/f1000research.11610.r19970

**Published:** 2017-02-06

**Authors:** Zhengyuan Xia

**Affiliations:** 1 Anesthesiology Research Laboratory, Department of Anaesthesiology, The University of Hong Kong (HKU), Hong Kong, China

I have read this submission. I believe that I have an appropriate level of expertise to confirm that it is of an acceptable scientific standard.

# Peer review: Referee response for version 2

**DOI:** 10.5256/f1000research.11302.r18941

**Published:** 2017-01-05

**Authors:** Amadou K.S. Camara

**Affiliations:** 1 Department of Anesthesiology, Medical College of Wisconsin (MCW), Milwaukee, WI, USA

**General comments**

 The manuscript “Inhibition of CD34+ cell migration by matrix metalloproteinase-2 during acute myocardial ischemia, counteracted by ischemic preconditioning” describes a phenomenon, preconditioning, that has been demonstrated to be cardioprotective against ischemic insult. The authors elegantly showed that the preconditioning that leads to protection involves the activation of CD34+ cell migration to the injured site for tissue remodeling, and that inhibition of MMP-2 is a potential target of the preconditioning protection. The details of the experimental protocols and procedures are adequate and the number of animals per treatment are statistically appropriate. The manuscript and the study design have some concerns that warrant the attention of the authors.

**Specific Comments**

Introduction

 The Introduction lacks a compelling rationale for why the in vivo studies were conducted in female pigs.

 The Introduction section does not make a distinction between early and delayed or second window cytoprotection conferred by preconditioning, and which of the two is being targeted in the current study. The concept of second window protection was discussed later in the manuscript; however, the reader should be provided this information earlier to orient and set the stage for an overview of the work.

 The last sentence in the Introduction “In addition, the current study explored a possible counteracting effect of IP on cytokines, CD34+ cell release and MMP-2 expression.” What does the “counteracting effect” mean in this case? Doesn’t this imply that IP diminishes the release of CD34+ cells, which are the necessary responses to protect against AMI during IP? Clarify or rephrase this sentence.

Materials

 Design of the porcine closed-chest reperfused acute myocardial infarction (AMI)

 The in vivo model lacks a sham group. This group should provide the proper basal level of the variables being monitored for the entire duration of the experimental protocol. The results shown in Figure 3 (3D) clearly attests and supports the inclusion of a sham group in this study.

 Isolation of human adult cardiomyocytes

 Were the cells obtained from male or female human subjects? If they are from male subjects, would the rationale for focusing on female pigs hold in this case? Or, does it not matter in this case?

 The purpose of using human cord blood CD34+ cells is not clear.

 Cell migration assay

 Figure 2, hypoxia treatment should have been present in all the various concentrations of SDF-1a and in its absence? This information would strengthen the connection between SDF-1a and the migratory response to CD34+ cells during hypoxia. How would one conduct an IP experiment in this case to demonstrate the restitution of the CD34+ migratory response during hypoxia in the in vitro model?

 The legend for Figure 2 (minor) “Lower and upper case chamber were then combined…” This should read, “Lower and upper case chambers were then combined…”

Results

 AMI-induced cytokine release and CD34+ mobilization

 “The circulating levels of the cytokines (FGF-2, VEGF, IL-8 and TNFa) were not changed at 3 days…” Where is the Figure/result that refer to this information? If there is none, please note in parenthesis (data not shown or displayed). What was the condition/treatment in this case?

 Based on the initial discussion of the results, the appearance of Figure 3B and Figure 3A should be switched.

 Cell migration

 Why was 90 min hypoxia used?

Discussion

 There seems to be a contradiction in the findings regarding the secretion of VEGF. As noted above in Results, there was no change in the levels of the cytokines (FGF-2, VEGF, IL-8 and TNFa) at day 3. In the Discussion, item 4, it notes “IP induced VEGF secretion in in the second window of cardioprotection.” Please clarify the discrepancy.

 Effect of AMI and IP PGF-2, VEGF, IL-8 and TNFa release

 The comparison being made in this section does not seem to account for the fact that the data were obtained from two experimental models and that the IL-8 was from pigs and not human cardiomyocytes.

 The conclusion could be bolstered by some of the control studies suggested.

I have read this submission. I believe that I have an appropriate level of expertise to confirm that it is of an acceptable scientific standard, however I have significant reservations, as outlined above.

# Response

**Published:** 2017-01-25

**Authors:** Dominika Lukovic

**Affiliations:** Medical University of Vienna, Austria

Dear Prof. Amadou K.S. Camara,

 We greatly appreciate that you spent a substantial amount of time looking at our paper entitled " Inhibition of CD34+ cell migration by matrix metalloproteinase-2 during acute myocardial ischemia, counteracted by ischemic preconditioning ".

**General comments: The manuscript “Inhibition of CD34+ cell migration by matrix metalloproteinase-2 during acute myocardial ischemia, counteracted by ischemic preconditioning” describes a phenomenon, preconditioning, that has been demonstrated to be cardioprotective against ischemic insult. The authors elegantly showed that the preconditioning that leads to protection involves the activation of CD34+ cell migration to the injured site for tissue remodeling, and that inhibition of MMP-2 is a potential target of the preconditioning protection. The details of the experimental protocols and procedures are adequate and the number of animals per treatment are statistically appropriate. The manuscript and the study design have some concerns that warrant the attention of the authors.**

****

**Specific comments:**

**Introduction**

**1A. Comment: The Introduction lacks a compelling rationale for why the in vivo studies were conducted in female pigs.**

Answer: Thank you for the comment. We agree with the reviewer in this aspect. Accordingly, we have corrected the Introduction section as follows (page 2):

 In our present experiment, we have investigated the mobilization of BM-origin CD34+ cells 3 days after reperfused AMI in relation to the SDF-1α/CXCR4 axis in a clinically relevant in vivo pig model. We conducted the experiments in domestic female pigs, since the female pigs tolerate the ischemic burden better regarding mortality as compared to male pigs ^22^ (page 2).

**

**1B. Comment:** **The Introduction section does not make a distinction between early and delayed or second window cytoprotection conferred by preconditioning, and which of the two is being targeted in the current study. The concept of second window protection was discussed later in the manuscript; however, the reader should be provided this information earlier to orient and set the stage for an overview of the work.**

Answer: We fully agree with this comment of the reviewer. We added the relevant information in the Introduction section as follows (page 2):

 Ischemic preconditioning-induced cardioprotection possesses bi-phasic effect by initiation of protective mechanisms in the early phase appearing within the first 3 hours following myocardial insult ^10 ^and in the late phase, which re-appear 24 hours and lasts for 72 hours ^16^ (Kamota et.al). In our study, we focused on the late window of cardioprotection in relation with stem cell mobilization and cytokine releases.

**1C. Comment:** **The last sentence in the Introduction “In addition, the current study explored a possible counteracting effect of IP on cytokines, CD34+ cell release and MMP-2 expression.” What does the “counteracting effect” mean in this case? Doesn’t this imply that IP diminishes the release of CD34+ cells, which are the necessary responses to protect against AMI during IP? Clarify or rephrase this sentence.**

Answer: Thank you for the comment. Indeed, we have rephrased the statement in the section Introduction (page 2):

 In addition, the current study explored a possible additional benefits elicited by IP that involve release of cytokines and CD34+ cells, and MMP-2 expression.

**Materials**

**2A.** **Comment:** **Design of the porcine closed-chest reperfused acute myocardial infarction (AMI). The in vivo model lacks a sham group. This group should provide the proper basal level of the variables being monitored for the entire duration of the experimental protocol. The results shown in Figure 3 (3D) clearly attests and supports the inclusion of a sham group in this study.**

Answer: Thank you for the important comment. We agree, that inclusion of a sham-operated control group would promote better comparison of the results. However, we decided to not supplement the study groups with control group of animals without infarction with expected constant baseline level of biomarkers. Indeed, we intended to apply the “reduction” principle of the 3R (reduction, refinement and replacement) concept regarding the in vivo experiments.

 We have added this comment in the Limitation (page 13).

**2B. Comment:** **Isolation of human adult cardiomyocytes. Were the cells obtained from male or female human subjects? If they are from male subjects, would the rationale for focusing on female pigs hold in this case? Or, does it not matter in this case?**

Answer: We thank the reviewer to highlight this issue. Isolation of human cardiomyocytes was performed from samples obtained from both female and male patients. Since the cells were utilized for *in vitro* experimental evaluation, the “gender” of the cells in the cell culture was not relevant for the in vivo experiments.

 We have added this comment in the Limitation (page 10) as follows:

 Isolation of human cardiomyocytes was performed from samples obtained from both female and male patients. Since the cells were utilized for in vitro experimental evaluation, the “gender” of the cells in the cell culture was not relevant for the in vivo experiments.

**2C. Comment:** **The purpose of using human cord blood CD34+ cells is not clear.**

Answer: We appreciate the reviewer’s opinion. We clearly noted the purpose of using human cord blood CD34+ cell in Method section (page 4):

 Human cord blood CD34 positive cells (CD34+ cells) were purchased from StemCell Technologies Company (Grenoble, France). The cells were used in *in vitro *cell migration assay to assess their migratory capacity toward HACMs. Since porcine CD34+ cells are not commercially available, we used human cardiomyocytes and human cord blood CD34+ cells.

**2.D Comment:** **Cell migration assay**

**Figure 2, hypoxia treatment should have been present in all the various concentrations of SDF-1a and in its absence? This information would strengthen the connection between SDF-1a and the migratory response to CD34+ cells during hypoxia. How would one conduct an IP experiment in this case to demonstrate the restitution of the CD34+ migratory response during hypoxia in the in vitro model?**

 Answer: Thank you for the valuable comment. SDF-1a treatment of the HACMs was conducted under normoxic conditions, since we wanted to assess the maximal mobilization effect of SDF-1a. We agree with the Reviewer that parallel measurements under hypoxic conditions might better explain the connection between SDF-1a and the migratory response.

 Ischemia and reperfusion stimuli of IP can be simulated *in vitro *by incubation of HACMs with alternation of normoxic and hypoxic conditioning cycles. This method was developed by Martou *et al.*

 Martou G, O'Blenes CA, Huang N, McAllister SE, Neligan PC, Ashrafpour H, Pang CY, Lipa JE (2006) Development of an in vitro model for study of the efficacy of ischemic preconditioning in human skeletal muscle against ischemia-reperfusion injury. Journal of applied physiology 101: 1335-42

**Results**

**3A. Comment:** **AMI-induced cytokine release and CD34+ mobilization**

**“The circulating levels of the cytokines (FGF-2, VEGF, IL-8 and TNFa) were not changed at 3 days…” Where is the Figure/result that refer to this information? If there is none, please note in parenthesis (data not shown or displayed). What was the condition/treatment in this case?**

Answer: Thank you for the remark. We have added this comment, and corrected the Result with the following text (page 5): 

 The circulating levels of the angiogenic cytokines (FGF-2, VEGF, IL-8 and TNFα) were not changed significantly at 3-days post-AMI in the AMI group (Figure 4A-D).

**3B. Comment:** **Based on the initial discussion of the results, the appearance of Figure 3B and Figure 3A should be switched.**

Answer: Thank you for the remark. Accordingly, we have corrected the Result section (page 5):

 Reperfused AMI did not enhance the release of SDF-1α at 3 days post-AMI (baseline: 32.02±24.35 vs. 3 days post-AMI: 26.97±15.43pg/ml; P=0.41) (Figure 3). In contrast, the circulating level of CXCR4 (baseline: 0.47±0.22 vs. 3 days post-AMI: 1.15±0.95ng/ml; P=0.034) increased significantly at 3 days post-AMI with concomitant induced mobilization of CD34+ cells (baseline: 260±75 vs. 3 days post-AMI: 668±180cells/μl; P<0.001).

**3C. Comment:** **Cell migration**

**Why was 90 min hypoxia used?**

Answer: The cells in *in vitro *cell migration assays were treated for 90 min, since 90 min LAD-occlusion was applied in the *in vivo *experimental protocol. *In vitro *experiment supposed to mimic the conditions applied in large animal experiment with already validated time for sufficient myocardial necrosis induced by 90 min coronary occlusion *(Gyöngyösi et. al, 2010).*

 Gyöngyösi M, Posa A, Pavo N, et al.: Differential effect of ischaemic preconditioning on mobilisation and recruitment of haematopoietic and mesenchymal stem cells in porcine myocardial ischaemia-reperfusion. *Thromb Haemost.* 2010;104(2):376–384. 20352158 10.1160/TH09-08-0558

**Discussion:**

**4A Comment:** **There seems to be a contradiction in the findings regarding the secretion of VEGF. As noted above in Results, there was no change in the levels of the cytokines (FGF-2, VEGF, IL-8 and TNFa) at day 3. In the Discussion, item 4, it notes “IP induced VEGF secretion in in the second window of cardioprotection.” Please clarify the discrepancy.**

Answer: Unchanged level of the FGF-3, VEGF and IL-8 and TNFa are related to the AMI group. We have corrected this in section Results (page 5). (Also Comment 3A).

**4B Comment:** **Effect of AMI and IP FGF-2, VEGF, IL-8 and TNFa release**

**The comparison being made in this section does not seem to account for the fact that the data were obtained from two experimental models and that the IL-8 was from pigs and not human cardiomyocytes.**

**The conclusion could be bolstered by some of the control studies suggested.**

 Thank you for the comment. We agree, the inclusion of the control group would give additional comparison to the group of the present experiments. However, our findings are similar to the study of Husebye *et al*., reporting no increase in TNF-alpha and IL-8 levels in patients with STEMI and randomized to placebo group.

 Husebye T1, Eritsland J2, Arnesen H3, Bjørnerheim R2, Mangschau A2, Seljeflot I4, Andersen GØ5. Association of interleukin 8 and myocardial recovery in patients with ST-elevation myocardial infarction complicated by acute heart failure. Plos One. 2014 Nov 12;9(11): e112359. doi: 10.1371/journal.pone.0112359. eCollection 2014

 We have added this comment in the Discussion (page 9).

 Additionally, we have expanded the Limitation section with the lack of sham-operated control group (page 10) as follows:

 Inclusion of a sham-operated control group would promote better comparison of the results. However, we decided to not supplement the study groups with control group of animals without infarction with expected constant baseline level of biomarkers. Indeed, we intended to apply the “reduction” principle of the 3R (reduction, refinement and replacement) concept regarding the in vivo experiments.

# Response

**Published:** 2017-01-30

**Authors:** Dominika Lukovic

**Affiliations:** Medical University of Vienna, Austria

**Comment:  Cell migration assay**

 Figure 2, hypoxia treatment should have been present in all the various concentrations of SDF-1a and in its absence? This information would strengthen the connection between SDF-1a and the migratory response to CD34+ cells during hypoxia. How would one conduct an IP experiment in this case to demonstrate the restitution of the CD34+ migratory response during hypoxia in the in vitro model?

**Answer: **Thank you for the valuable comment. SDF-1a treatment of the HACMs was conducted under normoxic conditions, since we wanted to assess the maximal effect of SDF-1a. We agree with the Reviewer that parallel measurement under hypoxic conditions might better explain the connection between SDF-1a and the migratory response.

 Ischemia and reperfusion stimuli of IP can be simulated *in vitro* by incubation of HACMS under normoxic and hypoxic conditioning cycles. This method was developed by Martou *et al*.

 Martou G, O'Blenes CA, Huang N, McAllister SE, Neligan PC, Ashrafpour H, Pang CY, Lipa JE (2006) Development of an in vitro model for study of the efficacy of ischemic preconditioning in human skeletal muscle against ischemia-reperfusion injury. Journal of applied physiology 101: 1335-42

# Peer review: Referee response for version 2

**DOI:** 10.5256/f1000research.11302.r17907

**Published:** 2016-12-20

**Authors:** Zhengyuan Xia, Michael Irwin

**Affiliations:** 1 Anesthesiology Research Laboratory, Department of Anaesthesiology, The University of Hong Kong (HKU), Hong Kong, China; 2 Department of Anesthesiology, University of Hong Kong, Pokfulam, K424, Hong Kong

Authors have adequately addressed reviewer concerns and improved the paper. No further comments.

We have read this submission. We believe that we have an appropriate level of expertise to confirm that it is of an acceptable scientific standard.

# Peer review: Referee response for version 1

**DOI:** 10.5256/f1000research.10732.r17966

**Published:** 2016-11-24

**Authors:** Zhengyuan Xia, Michael Irwin

**Affiliations:** 1 Anesthesiology Research Laboratory, Department of Anaesthesiology, The University of Hong Kong (HKU), Hong Kong, China; 2 Department of Anesthesiology, University of Hong Kong, Pokfulam, K424, Hong Kong

General comments: This is an interesting study with novel findings showing that myocardial ischemia triggers the release of circulating MMP-2, which inhibits SDF-1α and CXCR4 release, and that ischemic preconditioning (IP) inhibits MMP-2 release, thereby increasing both SDF-1α and CXCR4 levels, resulting in a higher level of CD34+ cell mobilization 3 days post ischemic injury in in vivo condition in large animals. In addition to the limitations mentioned by the authors, we have the following comments that the authors may need to consider to improve the manuscript.

 Specific comments:

1. Is there any particular consideration why female but not male pigs were chosen as the study subjects? This needs to be clarified. Similarly, the reason why n=6 in the IP group but n=12 in the non-conditioned AMI group is not clear.
2. IP has been shown to reduce post-ischemic IL-8 release in most, if not all, models of myocardial ischemia and reperfusion or in some clinical trial studies. While in your model, IP almost significantly increased, and could actually significantly (see below), post-ischemic IL-8. This should be discussed in comparison of other studies in more detail.
3. One may expect that IP may also could have significantly increased post-ischemic CXCR4, if the authors have 1) observed more time points; or 2) the sample size of the two groups were 9 per group rather than 6 vs. 12; or 3) the area under curve (AUC) data are compared. This applies to the above IL-8 story.
4. Have the authors considered to perform repeated measures ANOVA with post hoc comparison to assess time effects or time-treatment interaction?
5. The conclusion that IP enforces CD34+ cell mobilization via inhibition of MMP-2 does not seem to have well supported by data, in particular you did not have intervention of  MMP-2 in the presence or absence of IP in the in vivo model, and you did not measure cardiac MMP-2 proteins. Therefore, it needs to be toned down.

We have read this submission. We believe that we have an appropriate level of expertise to confirm that it is of an acceptable scientific standard, however we have significant reservations, as outlined above.

# Response

**Published:** 2016-12-15

**Authors:** Dominika Lukovic

**Affiliations:** Medical University of Vienna, Austria

Dear Dr. Zhengyuan Xia,

 We greatly appreciate the efforts to carefully review our paper and the valuable comments.

 We have modified the manuscript accordingly, as follows:

**General comments: This is an interesting study with novel findings showing that myocardial ischemia triggers the release of circulating MMP-2, which inhibits SDF-1α and CXCR4 release, and that ischemic preconditioning (IP) inhibits MMP-2 release, thereby increasing both SDF-1α and CXCR4 levels, resulting in a higher level of CD34+ cell mobilization 3 days post ischemic injury in in vivo condition in large animals. In addition to the limitations mentioned by the authors, we have the following comments that the authors may need to consider to improve the manuscript.**

**Specific comments:**

1. **A. Is there any particular consideration why female but not male pigs were chosen as the study subjects? This needs to be clarified. **

 Answer:

 Thank you for the comment. Indeed, we have chosen female pigs, because a clear gender differences have been observed in female and male rodents, rabbits, dogs and pigs (Murphy CVRes); the incidence of cardiogenic shock and life-threatening arrhythmias were more frequent in male than female pigs by using the closed-chest reperfused AMI model.

 We have added this comment into the Limitation in our revised paper (page…)

  

1. **B. Similarly, the reason why n=6 in the IP group but n=12 in the non-conditioned AMI group is not clear.**

 Answer:

 Due to expected higher mortality in the control group, the block randomisation of 1:2 was chosen. We have added this comment, and corrected the Method with the following text:

 Randomly selected female domestic pigs (n=21; weight, 30–35kg) underwent percutaneous coronary intervention (PCI) under general anaesthesia in order to perform either ischemic preconditioning (group IP) or non-conditioned AMI (group control), with a 1:2 block randomization, due to expected higher mortality in the control group. Animals in both groups underwent 90min percutaneous balloon occlusion of the left anterior descending (LAD) coronary artery at the origin of the first diagonal branch following reperfusion (balloon deflation). IP was initiated prior to 90min LAD occlusion by 3×5min repetitive cycles of artery re-occlusion and reperfusion. One pig in the IP and 2 animals in the control group died during the AMI intervention, all remaining animals (n=6 in IP and n=12 in control groups) survived for 1 month after the experimental procedure.

 (page 2)

**2. And 3. IP has been shown to reduce post-ischemic IL-8 release in most, if not all,  models of myocardial ischemia and reperfusion or in some clinical trial studies. While in your model, IP almost significantly increased, and could actually significantly (see below), post-ischemic IL-8. This should be discussed in comparison of other studies in more detail.** **One may expect that IP may also could have significantly increased post-ischemic CXCR4, if the authors have 1) observed more time points; or 2) the sample size of the two groups were 9 per group rather than 6 vs. 12; or 3) the area under curve (AUC) data are compared. This applies to the above IL-8 story.**

 Answer:

 Thank you for the remark. We agree that results of plasma cytokine levels would be more informative if it measured more often. However, an additional blood sampling would require a full anaesthesia in the pigs with recent AMI with expected higher mortality. Accordingly, we have added the following text in the revised manuscript:

 IL-8 is a pro-inflammatory C-X-X chemokine that is also involved in activation of pro-angiogenic processes and re-introduction of progenitor cells into the circulation. The study of Schomig et al. demonstrated significantly increased IL-8 level in AMI patients as compared to patients diagnosed with stable angina ^8^. In our study, we observed a trend toward increased release of IL-8 in the clinically relevant porcine reperfused “STEMI” model. The levels of CXCR4 increased both in controls (with AMI) and IP groups, with a trend towards higher increase in the IP group 3-day post AMI. The differences between our and other studies might be explained by the pre- and peri-AMI medication of patients with standard care that may contribute to changes in plasma levels of cytokines in AMI patients ^8^. Results of plasma cytokine levels would be more informative if it measured more often, in an extended time window. The area under the curve (AUC) calculation of the cytokine release data might have delivered additional results. However, for a simple blood sampling, the animals must have been fully anaesthesized, which procedure signifies an additional stress for the animals with recent AMI with predicted higher mortality.

 (page 9)

**4 Have the authors considered to perform repeated measures ANOVA with post hoc comparison to assess time effects or time-treatment interaction?**

 Answer:

 Thank you for the comment. Indeed, we have performed two-way ANOVA with repeated measurements, supplemented with Bonferroni correction, as described in the originally submitted paper (Statistical analysis section).

 According to the Reviewer`s suggestion, we have also tested the intra-group differences according to the time-factor (statistical comparison between baseline and 3-day and 1 month), and also the “between-groups” differences by using independent t-test. The results are added into the Figure legends as follows:

 +P<0.05  between  baseline  and  3  day  values  within  the  IP group;

  #P<0.05 between baseline and 3 day values within the control (non-conditioned  AMI) group. 

 *P<0.05 between  the IP and  control  group

 The non-significant changes were not labeled.

 We are aware, that serial blood sampling would have given more information, eg. the evident changes in mobilisation of bone marrow-derived cells following myocardial infarction occur at day 3, 7 and 14 post-ischemia ^23^ . However, we have focused on the second window of protection, which ends at day 3.

 We have added this comment in the Limitation (page 10).

**5. The conclusion that IP enforces CD34+ cell mobilization via inhibition of MMP-2 does not seem to have well supported by data, in particular you did not have intervention of MMP-2 in the presence or absence of IP in the in vivo model, and you did not measure cardiac MMP-2 proteins. Therefore, it needs to be toned down.**

 Answer:

 We agree with the reviewer in this aspect. Accordingly, we have corrected the Conclusion in the Abstract and Discussion and supplemented the Limitation section as follows:

 Abstract:

 Conclusions. Non-conditioned AMI induces MMP-2 release, hampering the ischemia-induced increase in SDF-1α and CXCR4 by cleaving the SDF-1α/CXCR4 axis, with diminished mobilization of the angiogenic CD34+ cells. IP might influence CD34+ cell mobilization via inhibition of MMP-2.

 (page 1)

 Discussion:

 IP induced CD34+ cell mobilization in the late phase (second window) of cardioprotection, thereby also increasing circulating SDF-1α and CXCR4, parallel with enhanced VEGF secretion. One mechanism of this beneficial effect of IP might be the inhibition of AMI-induced MMP2-release.

 (page 10)

 Limitation

 We revealed one additional possible beneficial mechanism of IP, namely the inhibition of MMP-2 release with consequent higher mobilization of CD34+ cells, which was confirmed in our in vitro experiment. However, a direct association between IP - MMP-2 - CD34+ axis had to be confirmed in vivo, by blocking MMP2 in animals subjected to AMI and IP. We have not measured myocardial MMP-2 level, which analysis would require harvesting of the animals max. 72h post IP-AMI (second window of protection), and our animals survived 1-month follow-up.

 (page 10)
